# Immediate surgical coronary revascularisation in patients presenting with acute myocardial infarction

**DOI:** 10.1186/1749-8090-8-167

**Published:** 2013-07-03

**Authors:** Nawid Khaladj, Dmitry Bobylev, Sven Peterss, Sabina Guenther, Maximilian Pichlmaier, Erik Bagaev, Andreas Martens, Malakh Shrestha, Axel Haverich, Christian Hagl

**Affiliations:** 1 Department of Cardiac, Thoracic, Transplantation and Vascular Surgery, Hannover Medical School, Carl-Neuberg-Str. 1, 30625 Hannover, Germany; 2 Department of Cardiac Surgery, University Hospital Munich, Marchionistr. 15, 81377 Munich, Germany

**Keywords:** Myocardial infarction, Coronary artery bypass grafting, Emergency surgery

## Abstract

**Background:**

The number of patients presenting with acute myocardial infarction (AMI) and being untreatable by interventional cardiologists increased during the last years. Previous experience in emergency coronary artery bypass grafting (CABG) in these patients spurred us towards a more liberal acceptance for surgery. Following a prospective protocol, patients were operated on and further analysed.

**Methods:**

Within a two year interval, 127 patients (38 female, age 68±12 years, EuroScore (ES) II 6.7±7.2%) presenting with AMI (86 non-ST-elevated myocardial infarction (NSTEMI), 41 STEMI) were immediately accepted for emergency CABG and operated on within six hours after cardiac catheterisation (77% three-vessel-disease, 47% left main stem stenosis, 11% cardiogenic shock, 21% preoperative intraaortic balloon pump (IABP), left ventricular ejection fraction 48±15%).

**Results:**

30-day-mortality was 6% (8 patients, 2 NSTEMI (2%) 6 STEMI (15%), p=0.014). Complete revascularisation could be achieved in 80% of the patients using 2±1 grafts and 3±1 distal anastomoses. In total, 66% were supported by IABP, extracorporal life support (ECLS) systems were implanted in two patients. Logistic regression analysis revealed the ES II as an independent risk factor for mortality (p<0.001, HR 1.216, 95%-CI-Intervall 1.082-1.366).

**Conclusions:**

Quo ad vitam, results of emergency CABG for patients presenting with NSTEMI can be compared with those of elective revascularisation. Complete revascularisation obviously offers a clear benefit for the patients. Mortality in patients presenting with STEMI and cardiogenic shock is substantially high. For these patients, other concepts regarding timing of surgical revascularisation and bridging until surgery need to be taken into consideration.

## Background

Multiple randomized studies have identified percutaneous coronary intervention (PCI) as being superior to medical thrombolysis in patients presenting with acute myocardial infarction (AMI) [1]. As a consequence, coronary angiograms are increasingly performed, resulting in a higher number of patients not suitable for PCI [2].

Nevertheless, primary coronary artery bypass grafting (CABG) in patients with three-vessel-disease is performed in only 5% of all patients presenting with AMI and in another 5% after primary PCI before discharge [3,4]. Similar results are seen in patients presenting with cardiogenic shock. In a recent study, only 3.5% of these patients underwent CABG during hospital stay [5].

Initial results in the 1970s showed dismal outcome for emergency CABG [6]. Within the last years, both perioperative management and myocardial protection improved significantly, leading to acceptable results of emergency CABG, even in patients with cardiogenic shock [7]. Due to our own experience on the one hand and that of other centers on the other, emergency CABG was not only considered as a rescue therapy anymore [8]. Therefore, we prospectively evaluated the outcome of an immediate surgical revascularisation strategy in patients presenting with acute myocardial infarction that were deemed untreatable or rejected by the referring cardiologist.

## Methods

This work was approved by the institutional review board of Hannover Medical School. All patients gave informed consent. The study was conducted at Hannover Medical School.

### Patients

Hannover Medical School is a tertiary care level I university hospital with a 24h helipad performing approximately 1800 cardiac procedures per year. From January 2009 to December 2010, 1333 patients underwent isolated CABG at our institution. Out of these, 127 (9.5%) patients presented with AMI (ST-segment elevation MI (STEMI) and non-ST-segment elevation MI (NSTEMI)) and underwent immediate CABG (less than six hours) after cardiac catheterisation. Patients were transferred to our department by ground or helicopter emergency service. Patients with sole instable angina were excluded from the analysis.

In addition to the demographic data, the EuroScore (ES) II was calculated for all patients. Patient’s demographics and preoperative data are depicted in Table 1. All patients were troponin positive. Troponin values are not presented since unfortunately cut off levels and standard measuring methods changed while the study was conducted.

**Table 1:** Demographics and preoperative data

**Variable**	**All patients (n=127)**	**NSTEMI (n=86)**	**STEMI (n=41)**	**p-value**
Age (years)	68±12	68±12	69±12	0.5
Female	38 (30%)	25 (29%)	13 (31%)	0.7
BMI (kg/m^2^)	28±5	28±5	27±4	0.3
Hypertension	95 (74%)	68 (79%)	27 (64%)	0.4
Current or ex-smokers	38 (30%)	29 (34%)	9 (21%)	0.2
Hyperlipoproteinemia	62 (48%)	45 (52%)	17 (40%)	0.5
Diabetes	27 (21%)	23 (27%)	14 (33%)	0.3
EuroScore II (%)	6.7±7.2	5.6±5.9	9.1±8.9	**0.023**
Previous MI	18 (14%)	12 (14%)	6 (14%)	0.9
Left main stem stenosis	59 (47%)	38 (44%)	21 (50%)	0.4
Three-vessel-disease	98 (77%)	69 (80%)	29 (70%)	0.2
LV-EF (%)	48±15	52±14	40±15	**0.001**
CPR	9 (7%)	4 (5%)	5 (12%)	0.1
Cardiogenic shock	14 (11%)	8 (9%)	6 (14%)	0.07
IABP	27 (21%)	16 (19%)	11 (26%)	0.3
CK (U/L)	558±669	511±647	665±714	0.3
CK-MB (U/L)	71±90	64±72	87±118	0.2
CK/CK-MB-Index (%)	14±9	14±10	13±9	0.6
CK-MB≥100 (U/L)	24 (19%)	19 (22%)	5 (12%)	0.06

*BMI* body mass index, *MI* myocardial infarction, *LV-EF* left ventricular ejection fraction, *CPR* cardio pulmonal resuscitation, *IABP* intra aortic balloon pump, *CK* creatin kinase, *CK-MB* MB isoenzyme of CK.

No special platelet function analyses have been performed in these patients.

### Preoperative and anesthesia management

The team consisted of a senior consultant anaesthesiologist, a senior consultant cardiothoracic surgeon, a senior resident and a cardiothoracic fellow. Additionally, an experienced scrub nurse and a perfusionist trained in the full spectrum of extracorporal support were available. If not onsite, team members were available within 30 minutes at all hours.

All patients had been rejected for interventional treatment by an experienced cardiologist of the referring hospital or department. In selected cases, an interventional cardiologist of our institution was consulted.

All patients were transferred to the operating room immediately upon arrival. In cases of delay, patients were transferred to the intensive care unit (ICU) and an intraaortic balloon pump (IABP) was inserted using a femoral access, according to the manufacturer’s suggestions. Blood pressure levels were kept high using vasopressors (e.g. norepinephrine) and the central venous saturation was kept above 60%. Monitoring was performed via a central venous line, an arterial line and a pulmonal arterial catheter in all patients.

Anesthesia was induced in a standard fashion; in patients with cardiogenic shock anesthetization took place in the operating theatre.

### Surgical technique

The detailed surgical technique is described elsewhere [8]. Briefly, after standard preparation surgery was performed using cardiopulmonary bypass and cardiac arrest (Buckberg cardioplegia) in most of the cases. A combination of left internal thoracic artery (ITA) and saphenous vein grafts was chosen for revascularisation whenever the patient was stable, according to the surgeon’s preferences. A left atrial catheter was obtained in all patients. An IABP was inserted if there were no contraindications.

### Postoperative management

Sedation was maintained until hemodynamic stabilisation was achieved. IABP was no contraindication for extubation. Blood transfusion and hemostatic therapy were adapted to chest drain output and laboratory parameters, as well as preoperative anticoagulation management. Patients that had been loaded with two platelet inhibitors received units of platelets after protamine administration in the majority of cases.

Heparin and aspirin (100 mg/d) were administered for anticoagulation within the first post-operative days. Later on, a dual antiplatelet therapy was implemented and continued for at least six months according to current guidelines [9].

### Statistical analysis

Results are given as mean ± standard deviation or percentage. Statistical analysis for group comparison was performed using the unpaired respectively paired t-test, the chi-square or Fisher’s exact test. Forward stepwise logistic regression analysis (likelihood ratio) was used to discriminate risk factors of 30-day mortality. The Kaplan-Meier survival estimate was used to analyse overall survival. Statistical differences were determined using the log-rank test. Receiver operating characteristic (ROC) was plotted: the area under the curve as well the sensitivity and specificity were calculated. A p-value of <0.05 was deemed significant. IBM SPSS Statistics (Vers. 20) software was used for statistical analysis.

## Results

Due to our previous experience and acceptance of this special cohort of patients, we had an increasing caseload of patients with AMI. Within this 2-year-interval, 127 patients were operated on. The number of patients not referred for surgery by the treating cardiologist as well as the detailed underlying reasons remain unclear, as well as the individual time delay between onset of symptoms and cardiac catheterisation.

### Preoperative data

**Figure 1:**
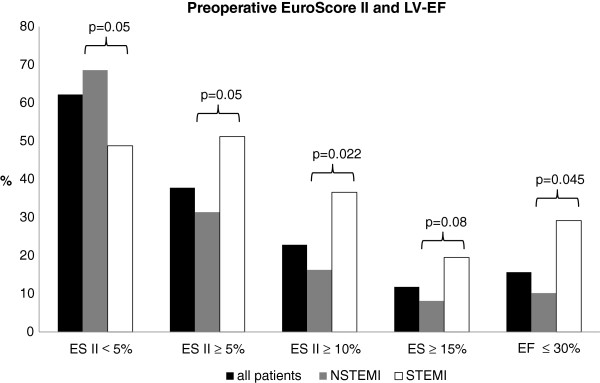
Patients were grouped according to Euroscore II (ES) and impaired left ventricular ejection fraction (LV-EF).

Patients were grouped according to etiology (NSTEMI/STEMI), EuroScore II as well as impaired left ventricular ejection fraction (LV-EF) (≤30%). The results of this comparison are plotted in Figure 1. All patients had been loaded preoperatively with intravenous aspirin (500 mg), a total of 41 patients (32%) additionally had received 600 mg clopidogrel per os.

### Intraoperative data

Intraoperative data are depicted in Table 2. Cardiopulmonary bypass and moderate hypothermia (34°C) were used in all patients with two or more vessel disease, in the majority of cases with antegrade administration of cold blood cardioplegia (Buckberg). On average, three anastomoses were performed (in 58% as a combination of an arterial graft and venous grafts) leading to a complete revascularisation rate of 80%. During reperfusion, another 57 IABPs were inserted. An extracorporal life support (ECLS) system was implanted in two patients.

**Table 2:** Intra- and postoperative data

**Variable**	**All patients (n=127)**	**NSTEMI (n=86)**	**STEMI (n=41)**	**p-value**
On Pump	120 (94%)	81 (94%)	39 (95%)	0.8
CPB (min)	85±30	83±26	89±38	0.3
Cross-Clamp (min)	37±16	38±17	37±15	0.9
Mammarian graft	74 (58%)	52 (60%)	22 (54%)	0.2
Number of grafts	2±1	2±1	2±1	0.5
Number of anastomoses	3±1	3±1	3±1	0.6
Complete revascularisation	102 (80%)	68 (79%)	34 (83%)	0.7
IAPB intraoperative	57 (45%)	40 (47)	17 (41%)	0.6
IABP total	84 (66%)	56 (65%)	28 (68%)	0.6
ECLS	2 (2%)	1 (1%)	1 (2%)	0.6
IAPB duration (d)	2±2	1±1	2±3	0.1
Postoperativ inotropic support	89 (70%)	59 (69%)	30 (73%)	0.8
CK/CK-MB-Index (%) 6 h	10±4	10±4	10±4	0.4
CK/CK-MB-Index (%) 24h	6±4	6±4	6±3	0.8
CK-MB≥100 (U/L) 6h	36 (28%)	19 (22%)	17 (62%)	**0.02**
CK-MB≥100 (U/L) 24h	20 (16%)	13 (15%)	7 (17%)	0.6
Respiratorory insuffiency	11 (9%)	6 (5%)	5 (12%)	0.3
Rethoracotomy	9 (7%)	5 (6%)	4 (10%)	0.4
ICU-stay (d)	5±6	4±4	5±8	0.2

*CPB* cardiopulmonary bypass, *IABP* intra aortic balloon pump, *ECLS* extracorporal life support, *CK* creatin kinase, *CK-MB* MB isoenyzme of CK, *ICU* intensive care unit.

### Postoperative data

Most of the patients (70%) needed inotropic support postoperatively. The index of cardiac markers decreased significantly (p<0.001) within the first day after surgery. The duration of IABP support was adapted to catecholomine dosage as well as the trend of cardiac markers. One patient of the STEMI group could not be weaned from IABP support and underwent subsequent implantation of a left ventricular assist device.

There was a slightly higher rate of re-exploration in the STEMI-group which however did not reach statistical significance. Receiving a loading dose of two platelet inhibitors was not a risk factor for re-exploration (p=0.1).

### Outcome

Temporary dialysis rate was low. Stroke rate was acceptable given the fact that none of the patients underwent evaluation of the carotid arteries preoperatively which is performed in routine patients. In patients with previous cardiopulmonary resuscitation (CPR) or that were intubated preoperatively, a detailed neurological evaluation was impossible (Table 3).

**Table 3:** Outcome

**Variable**	**All patients (n=127)**	**NSTEMI (n=86)**	**STEMI (n=41)**	**p-value**
Dialysis (new)	3 (2%)	1 (1%)	2 (5%)	0.2
Stroke	5 (4%)	5 (6%)	0	0.1
Hospital-mortality	7 (6%)	1 (1%)	6 (15%)	**0.005**
30-day-mortality	8 (6%)	2 (2%)	6 (15%)	**0.014**

### Survival

**Figure 2:**
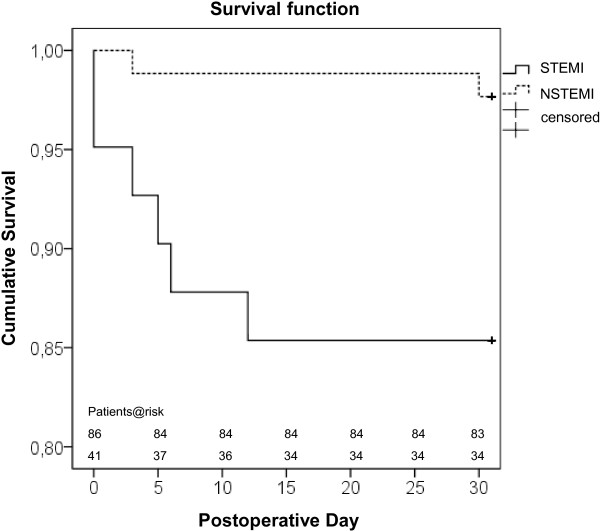
Kaplan-Meier Survival function, subdivided into NSTEMI and STEMI patients for the first 30 postoperative days.

Comparison of hospital as well as 30-day-mortality rates revealed significant differences between the NSTEMI and STEMI group. The Kaplan-Meier curve showed a significant better survival of the NSTEMI patients (p=0.007) (Figure 2). Furthermore, mortality in patients presented in cardiogenic shock preoperatively was significantly increased compared to those without shock (21% vs. 4%, p=0.043).

### Risk factor analysis for 30-day-mortality

Patients that died significantly more often had presented with a STEMI and in cardiogenic shock or were in need of IABP support preoperatively. LV-EF (29±17% vs. 50±14%) was reduced and EuroScore II values were increased (18±14% vs. 6±6%) (Table 4). Furthermore, CK-MB levels were more often >100 U six and 24 hours after revascularisation. These factors were used for a logistic regression model. The EuroScore II was the only independent predictor of mortality.

**Table 4:** Regression analysis (30-day-mortality)

**Factor**	**p**	**p (logistic regression)**
Cardiogenic shock	0.043	
IABP preoperative	0.064	
LV-EF	0.002	
STEMI	0.014	
CK-MB≥100 (U/L) 6h	<0.001	
CK-MB≥100 (U/L) 24h	0.054	
EuroScore II	0.04	**p<0.001**, HR 1.216, 95-CI-Intervall 1.082-1.366

*IABP* intra aortic balloon pump, *LV-EF* left ventricular ejection fraction, *STEMI* ST-elevated myocardial infarction, *CK-MB* MB isoenzyme of creatin kinase.

### ROC analysis for the EuroScore II

**Figure 3:**
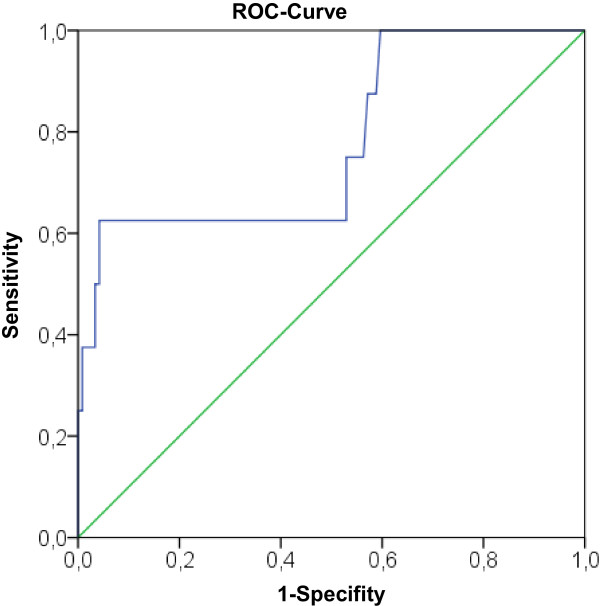
Receiver operating characteristics (ROC) curve for the EuroScore II.

A ROC curve was plotted for the EuroScore II (area under the curve 0.778) (Figure 3). The EuroScore II had 100% sensitivity (for an estimated risk up to 2.84%); specificity was 60%.

## Discussion

Mortality of patients with AMI undergoing emergency CABG could be reduced within the past years [2]. According to our own experience on the one hand and that of others on the other, survival could be improved using a prospective treatment protocol [10]. Therefore we changed our strategy for revascularisation of patients presenting with AMI [8]. Patients were accepted immediately from the referring cardiologist and were transferred to our emergency department. The sheath in the groin for cardiac catheterisation was kept in place for later on introduction of an IABP, if necessary. The patients were triaged by a board certified cardiothoracic surgeon and transferred immediately to the operating room. In cases of lacking diagnostics or shortage of capacity, the patient was transferred to the ICU and bridged with an IABP. In these cases, surgery was performed within the next 60 minutes.

Using this concept, the overall mortality rate was 6%. The mortality rate for NSTEMI patients was 2% which equals the general mortality rate for CABG in Germany in 2011 (2.9%) [11]. Mortality in the STEMI group (15%) was significantly increased in comparison to the NSTEMI patients, but lower than in our previous experience and that of others [7,8,12]. Even after failed PCI in patients with AMI and shock, CABG significantly improved survival [7]. In this study, the mortality rate could be reduced from 40.9 to 20.5% in the propensity score analysis. In the recent shock II trial, Thiele and co-workers performed a prospective study on the role of IABP usage for survival. Mortality rate was high in both groups. CABG was only performed in 3.5% of the patients despite the fact that more than 50% of the patients had a three-vessel disease [5].

Optimal timing is an important issue in this context. In a current analysis, Caceres and co-workers tried to elucidate the optimal timing of coronary artery bypass grafting in acute myocardial infarction [2]. They concluded that in general delaying CABG especially in hemodynamically stable patients cannot be considered as a standard of care anymore.

Excellent results were achieved by Alexiou and co-workers [12]. Mortality rate was less than 10% for NSTEMI as well as STEMI patients and immediate revascularisation was recommended. These results are to be interpreted in the context of an analysis from Assmann and associates [13]. In their retrospective analysis they identified early CABG as a risk factor for mortality and recommended at least a three-day waiting period. The rate of IABP support in patients that were revascularised immediately was low (10.5%) and the number of patients that died during the waiting interval remains unclear. This results in a decrease of the calculated mortality rate of patients that were revascularised later on [2].

According to current European guidelines, CABG is recommended three to seven days after AMI [14]. This recommendation is based on a retrospective study [15]. However, in patients with failed PCI or fibrinolysis and persistent hemodynamic instability, a IC recommendation is given for emergency surgery.

The EuroScore II might be useful to identify patients with a high operative risk in the future [16]. However, according to the data available so far ES II should not be used to reject patients since current studies revealed an unsatisfactory sensitivity and specificity. Furthermore, especially for these high risk patients no alternative revascularisation strategy exists.

The preoperative anticoagulation regime may have a further impact on surgical revascularisation. In 1998 clopidogrel entered the market leading to a decreasing number of studies addressing the issue of emergency CABG [2]. With the introduction of Ticagrelor and Prasugrel, two reversibly binding platelet inhibitors emerged in the recent year and may lead to further changes. For example, if Ticagrelor is stopped less than one day before surgery, 65.5% of the patients had CABG-related life threatening/fatal bleeding [17]. So far, clopidogrel was no contraindication for immediate revascularisation, but the recovery time of platelets after loading dose administration of prasugrel is up to nine days [18]. This may have an impact on further timing strategies.

Besides timing, the operative technique of revascularisation is controversially discussed. Rastan and co-workers recommended beating heart technique for patients presenting with acute coronary syndrome (including instable angina) [19]. However, they had a significantly lower rate of incomplete revascularisation in the beating heart group and short reperfusion times in the crossclamp group; the majority of patients received crystalloid instead of cold blood cardioplegia [20].

In our experience, reperfusion of at least 30 minutes is important in this patient cohort. This has been shown earlier in experimental as well as in clinical setups [21,22].

The number of mammary grafts should be further increased. However, the ITA was not used in patients presenting in cardiogenic shock or with high doses of vasopressors after induction of anesthesia, due to the fear of vasospasms occurring postoperatively [22,23].

In patients presenting in cardiogenic shock or with reduced LV-EF, the implantation of an extracorporeal life support system (ECLS) must be taken into consideration to achieve a longer reperfusion time and additional ventricular unloading [21,24,25]. If end-organ functions recover but weaning is impossible due to impaired LV-function, implantation of an left ventricular assist device is a feasible option, if no contraindication exists [26].

### Limitations

The number of patients presented is too low to establish an accurate estimation of the operative risk for this high risk patient cohort. The number of patients not referred for emergency revascularisation, especially those in cardiogenic shock, remains unclear.

There is no control group of patients that receive CABG after a waiting period, since in this two year interval all patients were operated on immediately. The same is true for patients receiving a PCI of the culprit lesion and CABG later on. Additionally, there is also no fitting control group given in current literature. Follow-up beyond 30 day is lacking so far.

## Conclusions

Immediate surgical revascularisation of patients presenting with acute AMI is feasible and leads to excellent results in NSTEMI patients. Mortality in STEMI patients, especially in those presenting in cardiogenic shock and or with impaired LV-function, is substantially high. For these patients, other timing or revascularisation strategies have to be implemented in the future. One option might be a more liberal implantation of an ECLS system in selected patients allowing myocardial recovery due to prolonged reperfusion and ventricular unloading. The same approach can be taken into consideration if myocardial markers do not decrease within the first 24 hours after surgery. Treating the culprit lesion only under ECLS support or ECLS standby, followed by CABG after stabilisation, might be another option. This has to be elucidated in further studies.

Nevertheless, not only treating the culprit lesion but achieving a complete revascularisation offers a clear advantage for the patient. This might be true especially for patients with three-vessel-disease and/or left main stem stenosis. Feasibility of immediate revascularisation of patients that have received a loading dose of novel, reversibly binding platelet inhibitor, has to be elucidate in further studies.

## Abbreviations

AMI: Acute myocardial infarction; CABG: Coronary artery bypass grafting; ES: EuroScore; NSTEMI: Non-ST-elevated myocardial infarction; STEMI: ST-elevated myocardial infarction; IABP: Intraaortic balloon pump; ECLS: Extracorporal life support; PCI: Percutaneous coronary intervention; ICU: Intensive care unit; ITA: Internal thoracic artery; ROC: Receiver operating characteristics; LV-EF: Left ventricular ejection fraction; CPR: Cardiopulmonary resuscitation; CK: Creatin kinase; CK-MB: MB isoenzyme of CK.

## Competing interests

The authors declare that they have no competing interests.

## Authors’ contributions

All authors have no financial or other interests regarding the submitted manuscript. NK, DB, SP and SG drafted the manuscript. NK, DB and SP performed statistical analysis. MP, EB, AM and MS collected the data and helped to draft the manuscript. AH and CH lead study design and coordination. All authors read and approved the final manuscript.
